# Supplementary material for: Dielectric Optical-Controllable Magnifying Lens by Nonlinear Negative Refraction
Source: Sci Rep. 2015 Jul 7;5:11892. doi: 10.1038/srep11892 (PMC4493703; doi:10.1038/srep11892)
Supplement: Supplementary Information [file srep11892-s1.pdf]

Dielectric Optical-Controllable Magnifying Lens

by Nonlinear Negative Refraction

Jianjun Cao[1], Ce Shang[2], Yuanlin Zheng[1] ,Yaming Feng, Xianfeng Chen[1,3], Xiaogan Liang[4] and Wenjie Wan[1,2,3*]

[1]Key Laboratory for Laser Plasmas (Ministry of Education), Department of Physics and Astronomy, Shanghai Jiao Tong University, Shanghai 200240, China

[2]University of Michigan-Shanghai Jiao Tong University Joint Institute, Shanghai Jiao Tong University, Shanghai 200240, China

[3]The State Key Laboratory of Advanced Optical Communication Systems and Networks, Shanghai Jiao Tong University, Shanghai 200240, China

[4]Department of Mechanical Engineering, University of Michigan, Ann Arbor, Michigan 48109, USA

Supplementary Information

1. Angle dependence for the nonlinear negative refraction in a flat medium

The scheme of the nonlinear negative refraction is depicted in Fig. S1(a). The pump beam at ω_1 is incident on a flat medium normally and the probe beam at ω_2 is incident at the angle of θ_2. The angle of the generated four wave mixing (4WM) wave in air is denoted as θ_3, measured with respect to the surface normal in counter-clockwise direction. The angles in air (θ_2 and θ_3) and the angles in the medium (θ_{2m} and θ_{3m}) are related by Snell's law:

$$\sin\theta_2 = n_2 \sin\theta_{2m}, \tag{1}$$

$$\sin\theta_3 = n_3 \sin\theta_{3m}. \tag{2}$$

To generate an efficient 4WM wave, phase matching condition, as shown in Fig. S1(b), should be satisfied, i.e.

$$\Delta \mathrm{k} = 2k_1 - k_2 - k_3 = 0, \tag{3}$$

where $k_i = \frac{2\pi n_i}{\lambda_i}$ $(i = 1, 2, 3)$ are the wave vectors of the pump, the probe and the 4WM beam respectively. The n_i are the corresponding refractive indexes of the medium. Decomposing Eq. (3) along k_z and k_x directions leads to

$$2\mathrm{k}_1 = \mathrm{k}_2 \cos\theta_{2m} + \mathrm{k}_3 \cos\theta_{3m}, \tag{4}$$

$$\mathrm{k}_2 \sin\theta_{2m} = -\mathrm{k}_3 \sin\theta_{3m}. \tag{5}$$

Inserting Eqs. (1) and (2) into Eq. (5), a Snell-like nonlinear refraction law is obtained:

$$\frac{\sin\theta_2}{\sin\theta_3} = -\frac{\lambda_2}{\lambda_3}. \tag{6}$$

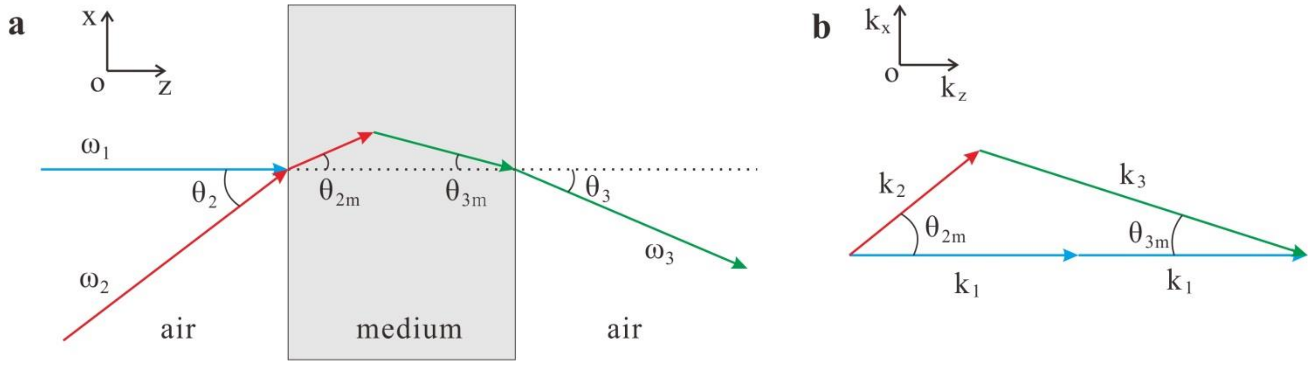

Figure S1 | Angle dependence. (a) Schematic of light paths of the pump, probe and 4WM beams. (b) Diagram of phase matching.

2. The nonlinear imaging law of a plano-concave lens

We consider that the four wave mixing process takes place in a plano-concave lens as shown in Fig. S2. The pump (ω_1) and probe (ω_2) beams enter the lens from the flat surface side so that the angle of the pump beam remains zero in the plano-concave lens, the same as the case in a flat medium. Then the 4WM beam (ω_3) is generated through nonlinear wave mixing process and the output angle is determined by nonlinear refraction law, shown as Eq. (6). The beam directions of the 4WM beams in the lens are shown as the dash lines in Fig. S2. At last, the 4WM beams are refracted by the concave surface and emit in directions shown as the green solid lines.

The whole process can be separated into two parts, firstly, a "virtual image" is generated by four wave mixing; secondly, the 4WM beams are refracted by the concave surface and form the "image". The virtual image distance "w" is determined from the object distance "u" by

$$\mathrm{w} = \mathrm{u} \cdot \frac{\tan\theta_2}{\tan\theta_3}. \quad (7)$$

The image distance "v" is related to the virtual object distance "w" by imaging rule:

$$\frac{1}{\mathrm{w}} + \frac{1}{\mathrm{v}} = \frac{1}{\mathrm{f}}, \quad (8)$$

which leads to

$$\mathrm{v} = \frac{\mathrm{f}\cdot\mathrm{w}}{\mathrm{w}-\mathrm{f}}. \quad (9)$$

Here we use sign convention that "u, v, θ_2" are positive and "w, f, θ_2"are negative. The image properties are governed by the relationship between "w" and "f": when $|\mathrm{w}| < |\mathrm{f}|$, the image is real, erect and magnified; when $|\mathrm{f}| < |\mathrm{w}| < 2|\mathrm{f}|$, the image is virtual, inverted and magnified; when

$2|f| < |w|$, the image is virtual, inverted and minified. If we want to obtain a real and magnified image, the object distance should be in the range $u < f \cdot \frac{\tan\theta_3}{\tan\theta_2}$. The magnification factor "M" can be calculated by

$$M_1 = \frac{v}{-w} = \frac{f}{f-w}. \quad (10)$$

Substituting Eq. (7) into Eq. (10), we obtain

$$M_1 = a \cdot \frac{v}{u} = \frac{af}{af-u}, \quad (11)$$

where $a = \frac{\tan\theta_3}{\tan\theta_2}$.

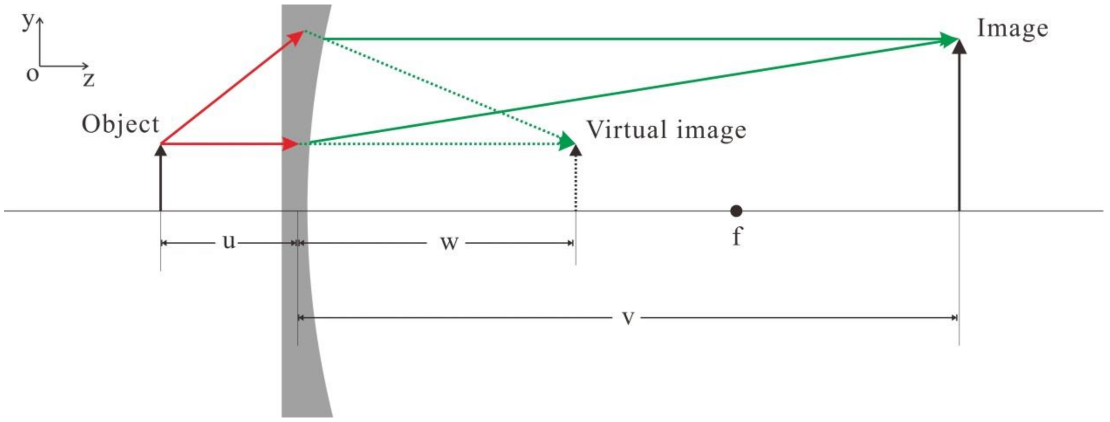

Figure S2 | Imaging behavior of the plano-concave lens. "u, w, v, f" are object distance, virtual image distance, image distance and focal length.

3. Non-collinear experimental setup for imaging by a plano-concave lens

The non-collinear experimental setup is depicted in Fig. S3. The pump and probe beams have the pulse duration of ~75 fs and repetition rate of 1 KHz. A delay line is added in the light path of the pump beam to ensure overlapping in time with the probe beam. A USAF resolution card, used as the "object", is placed on the probe's path, while the "image" formed with 4WM beams can be captured by a color CCD camera. The focal length of the plano-concave lens is -13.5 cm and its edge thickness is 1 mm.

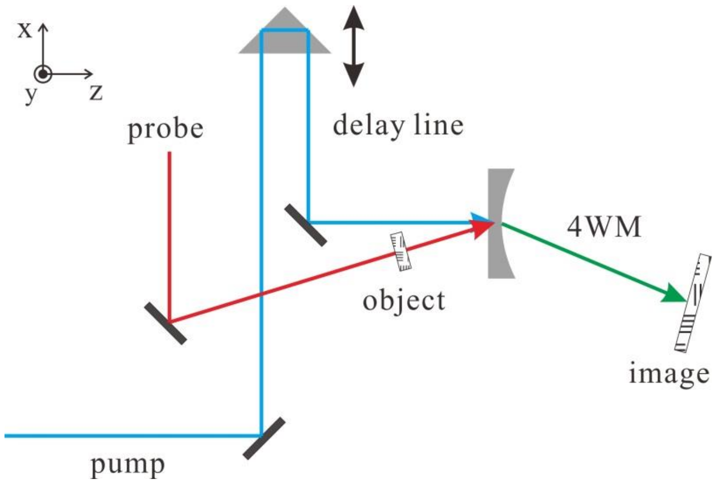

Figure S3 | Non-collinear experimental setup for imaging by a plano-concave lens

4. Phasing matching in *x-z* and *y-z* planes in non-collinear setup

In non-collinear experimental setup, phase matching is not isotropic in *x-z* and *y-z* planes. The phase matching condition ($\Delta \mathrm{k} = 2k_1 - k_2 - k_3 = 0$) requires that the wave vectors form a triangle in the wave vector space, as shown in Fig. S4(a). However, not all the input k_2, which contain the information of the object, can fulfill the requirement to generate 4WM beams. This causes the anisotropic imaging property in *x-z* and *y-z* planes. Projecting phase matching into the two planes respectively, we can find that all the probe beams can generate the same efficient 4WM beams in *y-z* plane, as shown in Fig. S4(b), which form clear horizontal structures in the image. However in *x-z* plane, just probe beams in the range of $\Delta\theta_2$ are possible to generate 4WM beams as shown in Fig. S4(c). Moreover, because the pump and probe beams are not exactly monochromatic, the 4WM beams are multicolor, giving rise to chromatic aberration. So the vertical structures in the image are blurry. The phase mismatching angle $\Delta\theta_2$ is determined by the thickness of the lens (*d*). Efficient 4WM can be generated when $\Delta \mathrm{k} \leq \frac{2\pi}{d}$. So by reducing the thickness of the lens, more probe beams can participate in the nonlinear process and the field of view will increase.

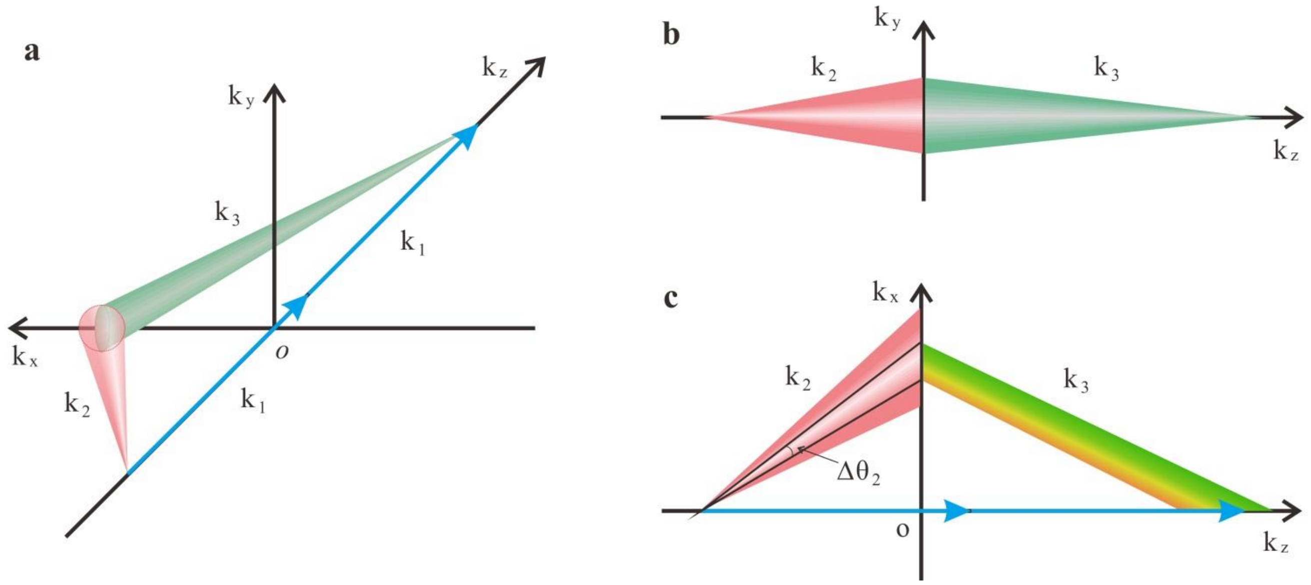

Figure S4 | Phase matching in non-collinear setup. (a) Diagram of phase matching in three dimension wave vector space. (b) Phase matching in *y*-*z* plane and (c) in *x*-*z* plane.

5. Collinear experimental setup for imaging by a plano-concave lens

The collinear experimental setup is depicted in Fig. S5. The pump beam at $\lambda_1 = 800\ nm$ is incident on the plano-concave lens normally, reflected by a dichroic mirror (900 nm long pass). The probe beam at $\lambda_2 = 1300\ nm$ modulated by a “grating” is transformed and forms an “object” in the front of the lens by a 4f system. The focal lengths of “L_1” and “L_2” in Fig. S5 are 4 cm and 6 cm, respectively. The zero order diffraction beam of the grating is blocked because this beam can’t fulfill phase matching. The focal length of the plano-concave lens used in this setup is -9.8 mm and its edge thickness is 1.98 mm. The “image” formed by the 4WM beam at $\lambda_3 = 578\ nm$ is recorded by a home build microscopy, made of a $40\times$ objective lens, a 600 nm short pass filter, a lens with focal length 15 cm and a high sensitive CCD camera.

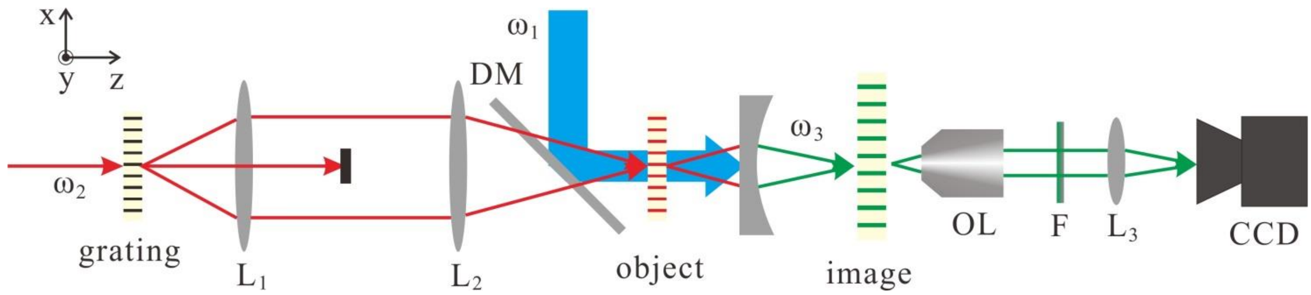

Figure S5 | Collinear experimental setup for imaging by plano-concave lens. L_1, L_2, L_3: lens; DM: dichroic mirror. OL: objective lens; F: filter.

6. The nonlinear imaging law of a flat lens with divergent pump beam

To turn a flat lens into a magnifying lens, the pump beam is changed from parallel light into spherical light. Considering that the pump and probe beams are incident at θ_1' and θ_2' as shown in Fig. S6(a). They are refracted by the front face of the flat lens to beams at θ_{1m}' and θ_{2m}'. Then the 4WM beam is generated in the lens through nonlinear process. The angle of the 4WM beam θ_{3m}' is determined by the phase matching condition as shown in Fig. S6(b). When θ_{1m}' is small, the angles have the relation

$$|\theta_{3m}'| = |\theta_{3m}| - \theta_{1m}', \tag{12}$$

where θ_{3m} is the angle of 4WM beam when the pump beam is incident normally. The relations between angles in air can be calculated by Snell' law:

$$\sin|\theta_3'| = n_3 \sin|\theta_{3m}'| = n_3 \sin(|\theta_{3m}| - \theta_{1m}') \approx n_3 \sin|\theta_{3m}| - n_3 \sin\theta_{1m}' = \sin|\theta_3| - \sin\theta_1' \quad . \tag{13}$$

Here, we use small angle approximation because θ_{3m} and θ_{1m}' is smaller than 5° in our experiments. With the above relations, the magnification factor of the flat lens with divergent pump beam can be calculated. The periods of the object (d_o) and the image (d_i) are related to the angles in air by

$$d_o = \frac{\lambda_2}{\sin\theta_2'} = -\frac{\lambda_3}{\sin\theta_3}, \tag{14}$$

$$d_i = -\frac{\lambda_3}{\sin\theta_3'}. \tag{15}$$

We obtain the magnification factor M_2:

$$M_2 = \frac{d_i}{d_o} = \frac{\sin\theta_3}{\sin\theta_3'}. \tag{16}$$

The imaging behavior of the magnifying flat lens is shown in Fig. S6(c), where "U, H, θ_1', θ_2'" are positive and "F, θ_3, θ_3'"are negative. We can write the angle of pump beam as

$$\tan\theta_1' = \frac{H}{-F} = \frac{U\cdot\tan\theta_2'}{-F}. \tag{17}$$

Inserting Eq. (13) and (17) into Eq. (16), we get

$$M_2 = \frac{1}{1-\frac{U}{F}\cdot\frac{\tan\theta_2'}{\tan\theta_3}}. \tag{18}$$

where small angle approximations ($\sin\theta_2' \approx \tan\theta_2'$ and $\sin\theta_3 \approx \tan\theta_3$) are used. This equation indicates that the image formed by the magnifying flat lens is not only determined from the object distance "U" but also from the focal distance "F", which makes it an optically controlled lens.

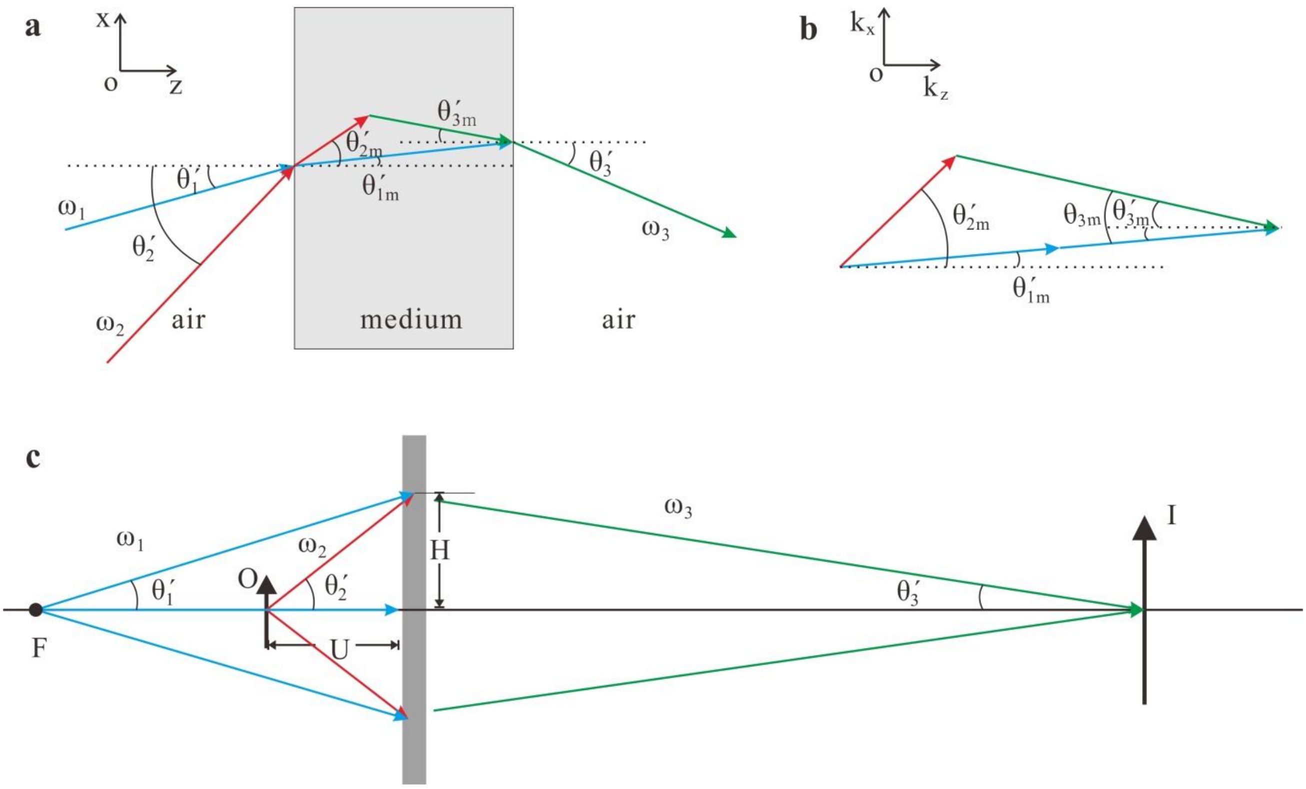

Figure S6 | Imaging behavior of the flat lens with divergent pump beam. (a) Schematic of light paths of the pump, probe and 4WM beams in the flat lens when the incident angle of the pump beam is not zero. θ_i' $(\mathrm{i} = 1, 2, 3)$ are angles in air and θ_{im}' are angles in the medium. (b) Phase matching triangle in the flat lens. θ_{3m} is the angle at the case that the pump beam is incident normally. (c) Imaging behavior of the flat lens with divergent pump beam. "O" and "I" stand for object and image. "F" is the distance between the divergent point of the pump beam and the lens. "U" and "H" are the object distance and the intercept of the probe beam on the lens.

7. Allowed angle spreading for 4WM generation

In strict phasing matching condition, 4WM wave can only be generated when the probe beam's angle θ_2 takes a specific value, determined by $\Delta k = 0$. However, in our experiments the pump and probe beams have multicolor spectrum with ~ 50 nm and ~ 100 nm line widths and the thin lens with 1 mm thickness allows nonlinear generation in the range $\Delta k \leq \frac{2\pi}{d}$. These two effects allow θ_2 to take values from $7.1°$ to $8.1°$. Experimentally measured 4WM intensities as a function of the probe beam's angle are shown in Fig. S7.

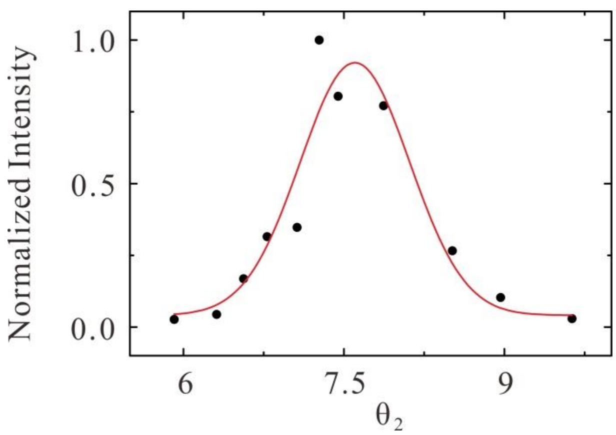

Figure S7. Measured 4WM intensity as a function of the probe beam's angle. The dots are experimentally measured value and the solid curve is a Gaussian fitting.
